# Skin Sensitization Study of Honey Bee (*Apis mellifera*) and Yellow Jacket *(Vespula* sp.*)* Venom on Rabbits

**Published:** 2019-08

**Authors:** Fatemeh BAGHERI, Mansoureh SHAYEGHI, Hassan VATANDOOST, Fatemeh NIKPOUR ALKARAN, Eslam MORADI ASL

**Affiliations:** 1. Department of Medical Entomology and Vector Control, School of Public Health, Tehran University of Medical Sciences, Tehran, Iran; 2. Department of Environmental Chemical Pollutants and Pesticides, Institute for Environmental Research, Tehran University of Medical Sciences, Tehran, Iran

## Dear Editor-in-Chief

Bee venom is a special and exceptional defensive weapon in the animal world. Bee venom is also the first and most important defense to defend the bee colony. Bee venom is a different combination of widest variety of chemical substances that protect the bees against a wide range of enemies (1). The most important allergen in bee venom composition is phospholipase A2, which is a glycoprotein with 134 amino acid residues (2). The main and great allergens in vespid venoms are phospholipase A1 (Ves v 1), hyaluronidase (Ves v 2), and antigen 5 (Ves v 5). Phospholipase A1 include 6–14% of the total dry weight of vespid venom (3). Antigen 5, is a considerable allergen in all vespid venoms (4). Skin sensitization potential plays an important part of any toxicology program for new consumer products (5).

For the purpose of skin sensation, we performed a toxicology performance on rabbits. We extracted honeybee venom and Wasp venom using existing methods. Totally 20 male rabbits were divided into 2 groups.

Ethical arrangement was performed by the Ethical Committee of Tehran University of Medical Sciences, Tehran, Iran.

All the experiments was carried out at laboratory of School of Public Health. One group were treated with wasp and honey bee sting with different dosage. In addition, we also tried to obtain the dilutions of the venom (3). Another 10 rabbits were tested using diluted venoms. The limit dose in this study was the level of 1,500 mg/kg BW. The details are presented in Tables 1 and 2. In each experiment, a rabbit was considered with distilled water treated as a control.

**Table 1:** Summary of clinical signs of dermal toxicity in rabbits immediately and 24 h after exposure to honey bee venom

	***Variable***	***Test site***								
***Group***	***Dose(mg/kg)***	***No. of animals***	***Inflammation Immediately after 24 h***		***Erythema Immediately after 24 h***		***Edema Immediately after 24 h***		***Death Immediately after 24 h***	
Vehicle control	0	2	0	0	0	0	0	0	0	0
Live honey bee sting	Different dosage	4	4	0	0	0	0	0	0	0
Purified honey bee venom	1,500 mg/kg	4	4	0	0	0	0	0	0	0

**Table 2:** Summary of clinical signs of dermal toxicity in rabbits immediately and 24 h after exposure to Wasp venom

***Variable***	***Test site***									
***Group***	***Dose (mg/kg)***	***No. of animals***	***Inflammation Immediately after 24 h***		***Erythema Immediately after 24 h***		***Edema Immediately after 24 h***		***Death Immediately after 24 h***	
Vehicle control	0	2	0	0	0	0	0	0	0	0
Live honey bee sting	Different dosage	4	4	4	0	4	0	2	0	0
Purified honey bee venom	1,500 mg/kg	4	4	4	0	4	0	4	0	0

Since honey bee venom has proven as antibacterial effects, it can be used to treat various types of ulcers (6). According to our study, it can be safely used in cosmetics and medical equipment. There is a classification for substances that have skin sensitivity to humans, based on that if there is evidence that the substance produces dermal complications in a significant number of humans, or responded positively to animal experiments (7).
